# Influence of Aerosol Delivered BCG Vaccination on Immunological and Disease Parameters Following SARS-CoV-2 Challenge in Rhesus Macaques

**DOI:** 10.3389/fimmu.2021.801799

**Published:** 2022-02-09

**Authors:** Andrew D. White, Laura Sibley, Charlotte Sarfas, Alexandra L. Morrison, Kevin Bewley, Colin Churchward, Susan Fotheringham, Konstantinos Gkolfinos, Karen Gooch, Alastair Handley, Holly E. Humphries, Laura Hunter, Chelsea Kennard, Stephanie Longet, Adam Mabbutt, Miriam Moffatt, Emma Rayner, Tom Tipton, Robert Watson, Yper Hall, Mark Bodman-Smith, Fergus Gleeson, Mike Dennis, Francisco J. Salguero, Miles Carroll, Helen McShane, William Cookson, Julian Hopkin, Sally Sharpe

**Affiliations:** ^1^Research and Evaluation, United Kingdom Health Security Agency, Salisbury, United Kingdom; ^2^National Heart and Lung Institute, Imperial College London, London, United Kingdom; ^3^Infection and Immunity Research Institute, St George’s University of London, London, United Kingdom; ^4^Department of Oncology, Churchill Hospital, Oxford, United Kingdom; ^5^The Jenner Institute, University of Oxford, Oxford, United Kingdom; ^6^College of Medicine, Institute of Life Science, Swansea University, Swansea, United Kingdom

**Keywords:** Aerosol BCG vaccination, SARS-CoV-2, COVID-19, macaque, cross-protection, non-specific, trained immunity

## Abstract

The tuberculosis vaccine, Bacille Calmette-Guerin (BCG), also affords protection against non-tuberculous diseases attributable to heterologous immune mechanisms such as trained innate immunity, activation of non-conventional T-cells, and cross-reactive adaptive immunity. Aerosol vaccine delivery can target immune responses toward the primary site of infection for a respiratory pathogen. Therefore, we hypothesised that aerosol delivery of BCG would enhance cross-protective action against severe acute respiratory syndrome coronavirus-2 (SARS-CoV-2) infection and be a deployable intervention against coronavirus disease 2019 (COVID-19). Immune parameters were monitored in vaccinated and unvaccinated rhesus macaques for 28 days following aerosol BCG vaccination. High-dose SARS-CoV-2 challenge was applied by intranasal and intrabronchial instillation and animals culled 6–8 days later for assessment of viral, disease, and immunological parameters. Mycobacteria-specific cell-mediated immune responses were detected following aerosol BCG vaccination, but SARS-CoV-2-specific cellular- and antibody-mediated immunity was only measured following challenge. Early secretion of cytokine and chemokine markers associated with the innate cellular and adaptive antiviral immune response was detected following SARS-CoV-2 challenge in vaccinated animals, at concentrations that exceeded titres measured in unvaccinated macaques. Classical CD14+ monocytes and Vδ2 γδ T-cells quantified by whole-blood immunophenotyping increased rapidly in vaccinated animals following SARS-CoV-2 challenge, indicating a priming of innate immune cells and non-conventional T-cell populations. However, viral RNA quantified in nasal and pharyngeal swabs, bronchoalveolar lavage (BAL), and tissue samples collected at necropsy was equivalent in vaccinated and unvaccinated animals, and in-life CT imaging and histopathology scoring applied to pulmonary tissue sections indicated that the disease induced by SARS-CoV-2 challenge was comparable between vaccinated and unvaccinated groups. Hence, aerosol BCG vaccination did not induce, or enhance the induction of, SARS-CoV-2 cross-reactive adaptive cellular or humoral immunity, although an influence of BCG vaccination on the subsequent immune response to SARS-CoV-2 challenge was apparent in immune signatures indicative of trained innate immune mechanisms and primed unconventional T-cell populations. Nevertheless, aerosol BCG vaccination did not enhance the initial clearance of virus, nor reduce the occurrence of early disease pathology after high dose SARS-CoV-2 challenge. However, the heterologous immune mechanisms primed by BCG vaccination could contribute to the moderation of COVID-19 disease severity in more susceptible species following natural infection.

## 1 Introduction

The emergence of the novel severe acute respiratory syndrome coronavirus-2 (SARS-CoV-2), the causative agent of coronavirus disease 2019 (COVID-19), instigated a worldwide pandemic that has resulted in more than four million deaths to date (1). As the pandemic evolved, regional and geographical differences in the incidence and severity of COVID-19 coincident with the use of the Bacille Calmette-Guerin (BCG) vaccine as part of national immunisation policy were identified (2–5). Currently, BCG delivered by intradermal (ID) injection is the only licensed tuberculosis (TB) vaccine and, despite a variable efficacy profile against adult pulmonary TB, remains widely administered in infancy due to the protection it affords against severe childhood forms of TB (6). In addition, BCG vaccination has been associated with non-specific protection against non-tuberculous childhood diseases and has been linked to a reduction in the incidence of all-cause childhood mortality and morbidity (7–9). These non-specific, cross-protective effects have been attributed to heterologous immune mechanisms such as trained innate immunity brought about by epigenetic reprogramming of monocyte and natural killer cell populations (10, 11), activation of non-conventional T-cells (12), and in the context of SARS-CoV-2, the induction of cross-reactive CD4+ and CD8+ T-cells responsive to homologous peptide sequences found in BCG and SARS-CoV-2 proteins (13). The potential for BCG-induced trained innate immune mechanisms to influence viral infections has been demonstrated in human challenge studies where the detection of epigenetic modifications within monocyte populations and the increased production of innate immune biomarkers including IL-1β, TNF-α, and IL-6 were associated with reduced yellow fever virus viraemia in recently BCG-vaccinated individuals (14). Consequently, the potential for BCG vaccination-induced cross-protection to influence the current and future pandemic situations has become a vibrant topic of debate (4, 15–17), and several clinical trials designed to determine the ability of ID BCG vaccination or revaccination to prevent SARS-COV-2 infection, or reduce the severity of COVID-19, are underway (18).

Although typically delivered as a single ID injection, BCG vaccination delivered *via* alternative routes has been shown to improve protection against TB (19–22). Aerosol delivery has been explored as a means to target vaccine-induced immune responses toward the primary site of infection at the respiratory mucosa (21, 23, 24) and is currently under investigation in ongoing clinical trials (25, 26). Recent reports have confirmed that the mucosal delivery of BCG leads to heightened induction of immune signatures associated with trained immunity (27); hence, we hypothesised that aerosol delivery of BCG *via* a portable vibrating mesh nebuliser (VMN) would enhance cross-protective action against infection with the respiratory pathogen SARS-CoV-2 and be a deployable intervention against COVID-19.

In this study, we have used an established rhesus macaque model (28–33) to explore the protective efficacy of aerosol-delivered BCG vaccination against experimental SARS-CoV-2 challenge and to profile a range of antigen-specific, and non-specific, immune parameters to explore the potential mechanisms of BCG vaccination induced cross-protection.

## 2 Results

### 2.1 Clinical Assessment Following Aerosol BCG Vaccination and SARS-CoV-2 Challenge

**Figure 1:**
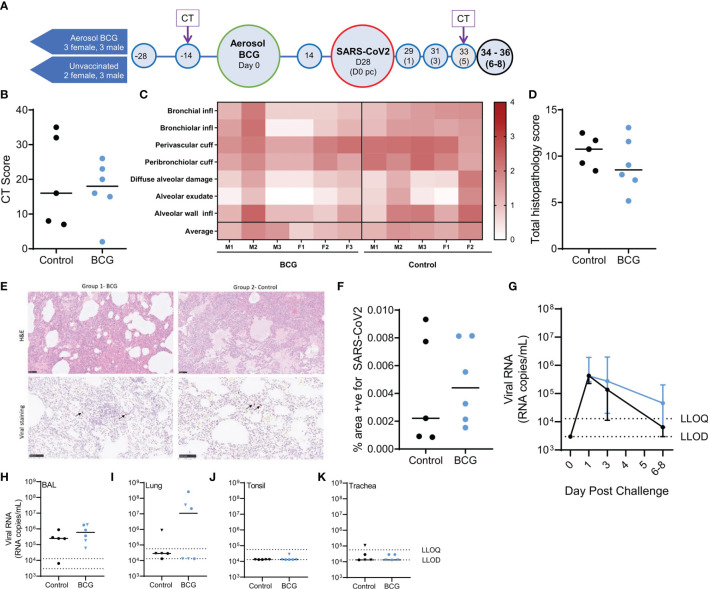
Study timeline relative to aerosol BCG vaccination and disease outcome measures following SARS-CoV-2 challenge. **(A)** Rhesus macaques received aerosol BCG vaccination (n = six) at study day zero or were left as unvaccinated controls (n = six, reducing to five after day 28). Animals received SARS-CoV-2 by split intranasal and intrabronchial challenge (target dose 5 × 10^6^ PFU) at day 28 and were monitored for up to 8 days post challenge (pc). Blue shaded circles represent procedures involving blood sample collection and application of immunological analyses; large circles represent key study events: vaccination and SARS-CoV-2 challenge, application of *in vivo* CT scanning is indicated. All animals were euthanized, and postmortem necropsies conducted upon completion of the study schedule (black circles) at days 34–36 (six–eight post challenge). **(B)** CT total score recorded at day five post-challenge. **(C)** Heatmap of pulmonary histopathology scores recorded in individual animals (males and females indicated). **(D)** Total lung histopathology scores. **(E)** Representative lung sections from animals from each group stained with either H&E or for expression of SARS-CoV-2 RNA. **(F)** Percentage area positive for SARS-CoV-2 staining in lung tissue sections. **(G)** SARS-CoV-2 viral RNA recovered from nasal swabs. Lower limits of detection (LLOD) and quantification (LLOQ) are indicated. **(H–K)** SARS-CoV-2 viral RNA recovered from **(H)** BAL samples collected at necropsy; **(I)** lung; **(J)** tonsil; and **(K)** trachea tissue samples. Plots show group median values (+/- IQR, plot G) with dots representing individual animals. Blue = BCG vaccinated, black = no vaccine.

Animals were observed and assessed for a range of clinical and behavioural parameters throughout the study (**Figure 1A**). Vaccinated animals were scored as active and healthy following aerosol BCG vaccination. Similarly, all vaccinated and unvaccinated animals were scored as active and healthy following SARS-CoV-2 challenge. Animal body weight was unperturbed by aerosol BCG vaccination and increased during the ensuing 4 weeks in all vaccinated animals. Weight loss was observed in unvaccinated control animals prior to SARS-CoV-2 challenge and in both BCG-vaccinated and unvaccinated animals following SARS-CoV-2 challenge (**Supplementary Figure 1**). One unvaccinated animal was removed from the study prior to SARS-CoV-2 challenge due to unrelated health issues. Immunological parameters recorded from this animal were included in comparative analysis of vaccine-induced immune profiles. The weight changes measured in aerosol BCG-vaccinated macaques following SARS-CoV-2 challenge was not significantly different to the weight loss observed in unvaccinated animals as determined by comparison of area under the curve values calculated from the post-challenge phase of the experiment (*p = 0.66*). Body temperature measurements were monitored throughout the study and remained within a normal range for all animals (**Supplementary Figure 2**). Blood haemoglobin concentration was measured at each blood sample collection procedure and declined progressively in both BCG-vaccinated and unvaccinated animals throughout the study time course due to repeated venesection (34) (**Supplementary Figure 3**).

### 2.2 Disease Pathology Induced by SARS-CoV-2 Challenge

Computed tomography X-ray (CT) scanning was performed prior to, and 5 days after, SARS-CoV-2 challenge and features of disease pathology assessed by a Consultant Radiologist blinded to animal treatment group and clinical status. Pulmonary abnormalities were identified on the CT scans collected from all BCG-vaccinated and unvaccinated control animals 5 days after SARS-CoV-2 challenge (**Figure 1B**). The disease burden measured as COVID pattern scores, zone scores, and total scores in the BCG-vaccinated group was similar to that measured in unvaccinated animals (**Figure 1B** and **Supplementary Figure 4**).

The following tissue sections were examined for histopathological changes: left cranial and caudal lung lobes (three sections from each), spleen, liver, kidneys (both), heart, colon, tonsil, larynx, trachea, and lung-associated lymph nodes. Histopathological changes were scored according to an established system (28), summarised in a heat map format in **Figure 1C**. The summed score for each parameter in the pathology scoring system is presented in **Figure 1D**, and representative images of lung parenchyma are shown in **Figure 1E**.

Microscopic changes observed in tissue samples were similar between aerosol BCG-vaccinated and unvaccinated animals (**Figures 1C–E**). Lesions observed in lung tissue samples were mainly inflammatory in nature and comprised variable numbers of mixed inflammatory cells, mainly neutrophils and lymphocytes, infiltrating bronchial and bronchiolar walls, with occasional epithelial degeneration and sloughing. More organised, focal lymphoid cell aggregates were noted with some frequency surrounding both large and small airways, as well as in perivascular locations. Less commonly, patchy, acute pneumonic changes were observed in the parenchyma. These comprised damage to alveolar cell walls and pneumocyte necrosis; alveolar spaces were filled variably with oedematous fluid, and variable fibrin, as well as mixed inflammatory cells (degenerate and non-degenerate neutrophils and macrophages). There was prominent thickening of alveolar walls by similar inflammatory cells. Low-grade inflammation of distal bronchioles and bronchiolo-alveolar junctions was noted variably, with epithelial degeneration and sloughing, admixed by neutrophils and macrophages. *In-situ* hybridisation staining (RNAscope) was applied to lung tissue sections for the detection of viral RNA (**Figure 1F**). Cells staining positive for SARS-CoV-2 RNA were observed rarely in inflammatory and alveolar cells in the lung of some aerosol BCG-vaccinated and unvaccinated animals; a difference in the frequency of staining between groups was not detected. Low-grade, non-specific, inflammatory changes were noted variably in the trachea, larynx, duodenum, colon, kidneys, and liver. These comprised mononuclear cell infiltration in the propria submucosa of the trachea and larynx, occasionally extending into tracheal glands; mild to moderate, lympho-plasmacytic cell infiltration of the lamina propria of the duodenum and colon; occasional lymphocytic cell foci in the renal cortical and medullary interstitium; and occasional, small foci of lymphocytes randomly located in hepatic parenchyma or surrounding portal triads. In the lung-associated lymph nodes, spleen, and tonsil, variable numbers of follicles with active germinal centres were noted. Changes were not observed in the heart.

### 2.3 Quantification of Viral Shedding, Viral Load, and Replication

Reverse transcription quantitative polymerase chain reaction (RT-qPCR) was used to monitor the presence of SARS-CoV-2 total viral RNA and sub-genomic (sg)RNA, in nasal and pharyngeal swab samples collected prior to and after SARS-CoV-2 challenge, and viral load was assessed in bronchoalveolar lavage (BAL) and tissue samples collected at necropsy.

Shedding of viral RNA was quantified in nasal swab samples collected from aerosol BCG-vaccinated and unvaccinated animals 1 day post SARS-CoV-2 challenge and declined steadily thereafter (**Figure 1G**). Comparison of AUC values indicated that nasal swab viral RNA titres were not altered by aerosol BCG vaccination in comparison to samples collected from unvaccinated animals (*p = 0.93*). Viral RNA was quantified in pharyngeal swab samples collected from three aerosol BCG-vaccinated animals, 3 days after SARS-CoV-2 challenge, whereas viral RNA was detected in pharyngeal swabs from one unvaccinated animal at this time point (**Supplementary Data 5**). Comparison of AUC values indicated that viral RNA titres quantified from pharyngeal swabs were not significantly different between the aerosol BCG vaccinated and unvaccinated group (*p = 0.27*).

SARS-CoV-2 RNA was detected in BAL samples collected postmortem from all aerosol BCG-vaccinated and unvaccinated animals (**Figure 1H**) with equivalent titres measured in each of the groups (*p = 0.43*). Viral RNA was detected and quantified in lung tissue samples collected from 50% of the aerosol BCG-vaccinated animals and detected in samples collected from 80% of the unvaccinated animals, although titres could only be quantified in one animal from this group (**Figure 1I**). The total viral RNA measured in lung samples collected from the aerosol BCG-vaccinated group was equivalent to that measured in the unvaccinated control group (*p = 0.83*). SARS-CoV-2 RNA could not be quantified in the tonsillar tissue samples collected from any animal, although viral RNA was detected above the LLOD of the assay in one aerosol BCG-vaccinated animal (**Figure 1J**). Similarly, viral RNA was detected in the tracheal tissue sample collected from two aerosol BCG-vaccinated animals and three unvaccinated macaques but only quantifiable in one unvaccinated animal (**Figure 1K**).

The presence of SARS-CoV-2 sgRNA was assessed in nasal and pharyngeal swabs and BAL and tissue samples as a measure of replicating virus (35, 36). Viral sgRNA was detected sporadically in swab samples and was detected, but could not be quantified, in BAL samples collected at necropsy from all animals, bar one unvaccinated macaque (**Supplementary Figure 5**). Copies of SARS-CoV-2 sgRNA were detected in lung tissue samples collected from three of the aerosol BCG-vaccinated animals, although quantification was only possible in two of these samples. Viral sgRNA was not detected in tracheal or tonsillar tissue samples (**Supplementary Figure 5**). Overall, the viral sgRNA copies measured in nasal or pharyngeal swabs and BAL or tissue samples collected from the aerosol BCG-vaccinated and unvaccinated groups were comparable.

### 2.4 Adaptive Immune Responses Induced by Aerosol BCG Vaccination and SARS-CoV-2 Challenge

#### 2.4.1 Cell-Mediated Immune Responses

Peripheral blood mononuclear cells (PBMC), lung mononuclear cells, and splenocytes were stimulated with a range of mycobacterial, SARS-CoV-2, and cytomegalovirus (CMV)-specific antigens to assess the cellular immune response to aerosol BCG vaccination and SARS-CoV-2 challenge.

**Figure 2:**
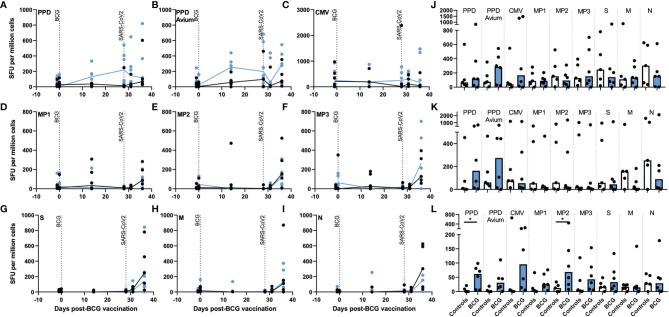
Antigen-specific IFN-γ producing cell frequency as detected by ELISPOT assay in PBMCs, lung MNC and splenocytes after aerosol BCG vaccination and/or SARS-CoV-2 challenge. Frequencies of **(A)** tuberculin PPD-, **(B)** PPD avium-, **(C)** CMV-, **(D)** spike protein MP1-, **(E)** spike protein MP2-, **(F)** spike protein MP3-, **(G)** spike complete peptide pool (S)-, **(H)** membrane (M)-, and, **(I)** nucleocapsid (N)-specific IFN-γ SFU measured before and after aerosol BCG vaccination or SARS-CoV-2 challenge. Antigen-specific IFN-γ SFU measured in **(J)** PBMCs, **(K)** lung MNCs and **(L)** splenocytes collected at necropsy. Black = unvaccinated control group, blue = aerosol BCG vaccinated. Medians shown. Mann–Whitney U tests were used for comparisons between groups (*p ≤* 0.05) and Wilcoxon matched pair test for comparisons of time points within groups (*p ≤* 0.05).

After aerosol BCG vaccination, the number of tuberculin PPD- and PPD avium-specific IFN-γ spot forming units (SFU) increased in the BCG-vaccinated group at days 14 and 28 post-vaccination (**Figures 2A, J**), with PPD-specific SFU frequencies significantly higher than in unvaccinated animals 28 days after vaccination (*p* = 0.030). These elevated tuberculin PPD-specific IFN-γ SFU were maintained after SARS-CoV-2 challenge, whereas PPD-avium-specific SFU decreased 3 days post challenge before increasing again at days 6–8 (**Figures 2B, J**). In contrast, mycobacterial antigen-specific IFN-γ SFU frequencies did not increase in samples collected from unvaccinated animals. In lung mononuclear cell and splenocyte samples (**Figures 2K, L**), there were more PPD- and PPD-A-specific SFU in the BCG-vaccinated group in comparison to samples collected from unvaccinated animals, a trend that reached significance for PPD-A-specific SFU measured in splenocytes (*p* = 0.0498).

To investigate whether aerosol BCG vaccination or SARS-CoV-2 challenge influenced the cellular immune response to viral pathogens in general, cytomegalovirus (CMV)-specific IFN-γ SFU were quantified. The presence of CMV-specific SFU was confirmed in all animals; however, there was no indication that either vaccination or challenge increased CMV-specific SFU in PBMC or lung MNC samples, although there were trends for more CMV-responsive cells to be detected in splenocyte samples collected from aerosol BCG vaccinated animals (**Figures 2C, J, K, L**).

Frequencies of SARS-CoV-2 spike peptide megapool: MP1-, MP2-, and MP3-specific IFN-γ SFU increased in PBMC samples collected from vaccinated and unvaccinated animals at necropsy, 6–8 days post SARS-CoV-2 challenge (**Figures 2D, E, F, J**), although frequencies were comparable between the aerosol BCG-vaccinated and unvaccinated groups. Low levels of MP-specific IFN-γ-secreting cells were observed in MNC cells isolated from lung tissue samples (**Figure 2K**), whereas in the spleen, MP-specific IFN-γ SFU were higher in BCG-vaccinated animals, significantly so for MP2 (*p* = 0.0498) (**Figure 2L**). Spike- (S), membrane- (M), and nucleocapsid- (N) complete peptide pool-specific SFU also increased in samples collected at necropsy from both groups (**Figures 2G–I**), although there were trends for S- and N-peptide-specific IFN-γ SFU frequencies to be greater in PBMC samples collected from unvaccinated animals (**Figure 2J**). Similarly, there were trends for SARS-CoV-2 M- and N-specific SFU to be greater in lung MNCs collected from unvaccinated animals (**Figure 2K**), whereas in the spleen, IFN-γ SFU frequencies measured in aerosol BCG-vaccinated and unvaccinated animals were comparable (**Figure 2L**).

#### 2.4.2 Humoral Immunity

Titres of anti-spike protein, anti-spike receptor-binding domain (RBD), and -nucleocapsid protein (NP) IgG were quantified before and after aerosol BCG vaccination and SARS-CoV-2 challenge by ELISA assay. No appreciable increase in IgG titres were detected following aerosol BCG vaccination or SARS-CoV-2 challenge in either experimental group (**Supplementary Figure 6**). Neutralising antibodies were quantified using microneutralisation assays (37) but were not detected prior to, or following, aerosol BCG vaccination (**Supplementary Figures 7A–C**). Titres increased in both aerosol BCG-vaccinated and unvaccinated control animals from 6 days following SARS-CoV-2 infection. Comparison of AUC values calculated from neutralising antibody titres quantified following SARS-CoV-2 challenge (Days 28–35 post BCG) indicates that neutralising antibody production did not differ between the aerosol BCG-vaccinated and unvaccinated animals (*p* = 0.62) (**Supplementary Figure 7**).

### 2.5 Whole-Blood Immunophenotyping

**Figure 3:**
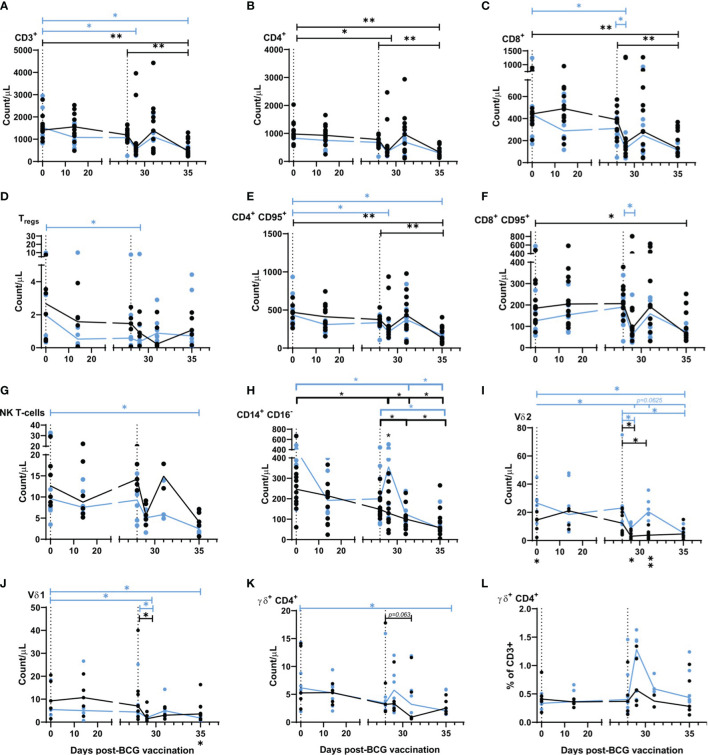
Immunophenotyping applied to whole blood before, and after aerosol BCG vaccination and SARS-CoV-2 challenge. **(A)** CD3+ T-cells, **(B)** CD4+ T-cells, **(C)** CD8+ T-cells, **(D)** Tregs, **(E)** CD4+ CD95+ T-cells, **(F)** CD8+ CD95+ T-cells, **(G)** NK T-cells, **(H)** CD14+ CD16+ monocytes, **(I)** Vδ2 cells, **(J)** Vδ1 cells, **(K)** γδ+ CD4+ cells, **(L)** γδ+ CD4+ cells as a proportion of CD3+ cells. All cell populations shown as counts/µl. Group medians are shown with data from individual animals represented by a dot. Blue = BCG vaccinated (n = six), black = unvaccinated (n = 12 or six, reducing to 11 and five post challenge). Significant differences determined by the Wilcoxon signed-rank test for comparisons within groups (colour coded by group) and Mann–Whitney U-test for comparisons between groups are denoted by asterisks. *p ≤ 0.05, **p = 0.01.

A whole-blood immunophenotyping assay was applied at regular intervals before and after aerosol BCG vaccination and SARS-CoV-2 challenge to quantify and determine the dynamics of leukocyte populations. T-cell populations CD3+, CD4+, and CD8+ all decreased from 1 day after SARS-CoV-2 challenge in BCG-vaccinated and naïve animals (**Figures 3A–C**), with CD3+ T-cell counts measured at day 1, 3, and 6–8 post SARS-CoV-2 challenge significantly lower in aerosol BCG-vaccinated animals in comparison to pre-vaccination levels (all *p* = 0.0313). CD8+ T-cell counts measured 1 day post challenge were significantly lower in comparison to pre-vaccination, and the day of SARS-CoV-2 challenge (DOC) in aerosol BCG-vaccinated animals (both *p* = 0.0313) (**Figure 3C**). Regulatory T-cells (Tregs) decreased in naïve animals, 3 days after SARS-CoV-2 challenge, but recovered to DOC levels by the end of the study (**Figure 3D**). In the aerosol BCG-vaccinated group, Treg counts were significantly lower than measured at baseline, 1 day post challenge (*p* = 0.0313), but had increased when quantified 2 days later. Counts of memory T-cells, as defined by CD95+ expression, changed after infection in both groups (**Figures 3E, F**). CD4+ CD95+ T-cells decreased in the aerosol BCG-vaccinated group in comparison to baseline values at days 1 and 6–8 post SARS-CoV-2 challenge (both *p* = 0.0313), and a non-significant decrease was observed in the unvaccinated animals at days 6–8 post challenge in comparison to baseline and DOC values. CD8+ CD95+ T-cell counts also decreased 1 day after challenge in the aerosol BCG-vaccinated group in comparison to DOC (*p* = 0.0313).

Non-significant decreases in the NK T-cell population were measured 1 day post SARS-CoV-2 challenge in both groups, although counts increased again by day 3 in unvaccinated animals, in contrast to a steady decline observed in the aerosol BCG-vaccinated group (**Figure 3G**) which culminated in levels of NK T-cells being significantly lower than pre-vaccination values in the BCG-vaccinated animals at days 6–8 post challenge (*p* = 0.0313).

Classical monocyte populations (CD14+ CD16-) decreased steadily throughout the study in unvaccinated animals (**Figure 3H**), whereas, in the aerosol BCG-vaccinated group, a rapid increase in classical monocyte counts was measured 1 day after SARS-CoV-2 challenge. Monocyte counts measured at day 3 and days 6–8 post challenge were significantly different to baseline values (day 3 *p* = 0.0313, days 6–8 *p* = 0.0313) and counts recorded on the DOC in the aerosol BCG-vaccinated group (*p* = 0.0313).

γδ T-cell subtypes Vδ1, Vδ2, and γδ+ CD4+ T-cells were also monitored (**Figures 3I–L**). There were significantly lower Vδ1 and Vδ2 T-cell counts in the blood 1 day after SARS-CoV-2 challenge, although there was evidence of a transient recovery in Vδ2 counts in the blood 3 days post challenge that led to significant differences in Vδ2 counts between the vaccinated and unvaccinated groups (*p* = 0.0426 at day 1 and *p* = 0.0027 at day 3 post challenge). A trend for increased γδ+CD4+ cell counts was noted 1 day after challenge in aerosol BCG-vaccinated animals (**Figure 3K**), and when this was expressed as a proportion of CD3+ cells, there was a significant increase in the proportion of γδ+ CD4+ T-cells (*p* = 0.0313) (**Figure 3L**). Correlations between whole blood counts of γδ T-cell subtypes and disease outcome measures were investigated (**Supplementary Figure 8**). A higher Vδ2 count 1 day post challenge correlated with lower bronchiolar inflammation (r = -0.6299, *p* = 0.0424), alveolar exudate (r = -0.6119, p = 0.0498), and peri-bronchiolar cuffing (r = -0.5895, *p* = 0.0606). Conversely, higher Vδ1 counts on the day of challenge correlated with higher CT scores and diffuse alveolar damage (r = 0.8565, *p* = 0.0013 and r = 0.6256, *p* = 0.0438), whereas trends for γδ+ CD4+ T-cells to correlate with a lower average histopathology score (r = -0.6059, *p* = 0.0527) and CT total score (r = -0.5616, *p* = 0.0757) at days 6 –8 post challenge were also identified. Changes in the phenotype of the Vδ2 population were detected after SARS-CoV-2 challenge (**Supplementary Figure 9**) with the proportion of Vδ2 cells expressing the early activation marker CD69 significantly elevated in both groups between 1 and 3 days post challenge (*p* = 0.0313, BCG group, *p* = 0.0391, unvaccinated controls) and decreased in the BCG group at necropsy (*p* = 0.0323). There was a trend toward a reduction in Vδ2 expression of exhaustion markers PD-1 and TIM3 in the BCG group, and an increase in their expression in the unvaccinated animals. Differences in memory phenotype of Vδ2 cells were also evident after challenge, with the BCG group having a higher proportion of central memory cells (*p* = 0.0303, 1 day post challenge, *p* = 0.0023, and 3 days post challenge) and a lower proportion of naïve cells (*p* = 0.0223, 3 days post challenge) than the unvaccinated control group.

### 2.6 Quantification of Secreted Biomarkers in Serum

**Figure 4:**
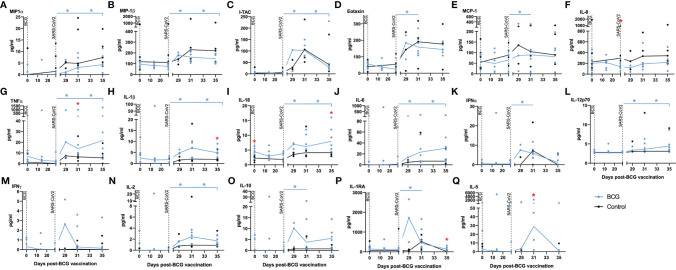
Chemokine markers in serum by multiplex bead array assay. Plots show titres of selected chemokine markers measured in serum samples collected from individual animals with group median values plotted as lines. **(A)** MIP-1α, **(B)** MIP-1β, **(C)** I-TAC, **(D)** eotaxin, **(E)** MCP-1, **(F)** IL-8, **(G)** TNF-α, **(H)** IL-1β, **(I)** IL-18, **(J)** IL-6, **(K)** IFNα, **(L)** IL-12p70, **(M)** IFN-γ, **(N)** IL-2, **(O)** IL-10, **(P)** IL-1RA, **(Q)** IL-5. Aerosol BCG vaccination and SARS-CoV-2 challenge are indicated by dotted lines. Asterisks indicate significant differences between pre- and postinfection values (colour coded to vaccination group) measured by the Wilcoxon matched-pair test. Red asterisks indicate significant differences between the groups determined by the Mann–Whitney U-test (*p ≤* 0.05).

Serum collected from the macaques was evaluated for the presence of 30 cytokines, chemokines, and growth factors to identify vaccine-, or challenge-, specific responses. Of the 30 analytes measured, the concentrations of 13 (G-CSF, GM-CSF, IL-13, IL-15, IL-17A, IL-23, IL-4, IL-7, IP-10, SDF-1alpha, MIG, CD40-ligand, BLC) were either undetectable (below the limit of detection of the assay system) or did not differ between the test groups. Significant differences in analyte titres were not detected between aerosol BCG-vaccinated and unvaccinated animals prior to, or following, aerosol BCG vaccination. Titres of the chemokines MIP-1α, MIP-1β, I-TAC, eotaxin, and MCP-1 (**Figures 4A–E**) were significantly increased (all *p* = 0.0313) after challenge with SARS-CoV-2 in the BCG-vaccinated group, although there was a trend for higher concentrations of these chemokines, and IL-8 (**Figure 4F**), to be detected in the unvaccinated group compared to the BCG-vaccinated animals following SARS-CoV-2 challenge.

Pro-inflammatory cytokines TNF-α, IL-1β, IL-18, and IL-6 (**Figures 4G–J**), which are associated with trained immune responses, increased significantly following SARS-CoV-2 challenge in BCG-vaccinated animals (all *p* = 0.0313) and were detected at higher concentrations than in unvaccinated animals (*p* = 0.05). Cytokines associated with initiation of the antiviral and adaptive response, IFNα, IL-12p70, IFN-γ, and IL-2 (**Figures 4K–N**), were detected earlier in BCG-vaccinated animals and increased significantly in BCG-vaccinated animals following SARS-CoV-2 challenge (all *p* = 0.0313).

Concentrations of the immunomodulatory cytokines IL-10 and IL-1RA (**Figures 4O, P**) were significantly increased (both *p* = 0.0313) in BCG-vaccinated animals following SARS-CoV-2 challenge. There were also significantly higher concentrations of the anti-inflammatory cytokine IL-5 (**Figure 4Q**) in BCG-vaccinated macaques 3 days after SARS-CoV-2 challenge compared to titres measured in unvaccinated macaques (*p* = 0.015.

## 3 Discussion

Since the outbreak of SARS-CoV-2 infections and rapid global increase in cases of COVID-19 witnessed during 2020, the trajectory of the pandemic has been changed significantly by the development, regulatory approval, and deployment of highly effective SARS-CoV-2-specific vaccines. To date, at least eight novel vaccines have been approved for emergency use by various international regulatory committees following the completion of clinical trials demonstrating favourable safety profiles and efficacy in reducing the occurrence of COVID-19 disease (38). The impressive pace at which these SARS-CoV-2-specific vaccines have become available has inevitably reduced the focus on BCG as a deployable intervention or therapeutic to aid control of the COVID-19 pandemic. However, as a non-specific treatment with the capacity to provide broad-spectrum immunological influence, BCG may still have relevance to the current, and more importantly, future pandemic situations.

At the time of writing, there are at least 20 clinical trials underway designed to interrogate the potential benefits of intradermally delivered BCG vaccination, or revaccination, as a strategy to protect against SARS-CoV-2 infection or to reduce the severity of COVID-19 disease (18). The findings of these trials, and particularly the randomized controlled clinical trials, are eagerly awaited and will provide the necessary evidence to determine whether BCG vaccination is able to provide protective efficacy against SARS-CoV-2 infection and the subsequent development of disease, or has other beneficial therapeutic properties relevant to the control of the current pandemic. For example, if clinical benefit can be demonstrated, a low-cost, widely available, and easy-to-administer product such as BCG could be a valuable countermeasure in regions where supply and infrastructure barriers restrict the rollout of SARS-CoV-2-specific vaccines (7). Furthermore, the broad-spectrum immunological influence of BCG may help to minimise the emergence of SARS-CoV-2 strains resistant to specific-vaccine-induced immunity through the activation and recruitment of non-conventional cellular immunity (12) and have adjuvant properties when administered alongside SARS-CoV-2 vaccines aiding the development of long term immune memory (39, 40). However, the scaling of production and supply required for BCG to be deployed as an intervention against non-tuberculous diseases would need to be carefully managed and coordinated to avoid compromising the provision of vaccine for the ongoing TB eradication campaign.

We, and others, have previously demonstrated that aerosol-delivered BCG vaccination is safe and capable of generating potent mucosal and systemic antigen-specific immune responses (20, 23, 24), and direct pulmonary delivery of BCG has recently been shown to be a superior method for the induction of trained innate immune mechanisms in comparison to parenteral vaccination in rhesus macaques (27). Therefore, in this experiment, we hypothesised that aerosol BCG vaccination would lead to cross-protective effects in a rhesus macaque SARS-CoV-2 experimental challenge model and provide an opportunity for the mechanisms underpinning this protection to be explored.

Developed and optimised during the initial stages of the 2019 pandemic for rapid screening of SARS-CoV-2-specific vaccine candidates, the rhesus macaque SARS-CoV-2 model employs a stringent high-dose challenge protocol whereby a combined 5 × 10^6^ PFU is delivered intranasally and by intrabronchial instillation, followed by measurement of viral shedding, tissue viral load, and early disease pathology readouts during the initial 6–8-day following challenge (28, 32). To date, this model has proved useful in the assessment of several SARS-CoV-2-specific vaccines (29–31, 33), although it is evident that the viral readouts and pathological changes observed in macaques do not fully reflect the characteristic features of severe COVID-19 seen in susceptible human patients (32). The findings of our experiment align with this view as we observed few perturbations in clinical parameters following SARS-CoV-2 challenge in either aerosol BCG-vaccinated or unvaccinated macaques, thereby demonstrating that the clinical symptoms observed in patients afflicted with severe COVID-19 are not replicated in this species. Where mild changes were observed, such as in body weight, blood haemoglobin concentration, and body temperature, these were comparable between the aerosol BCG-vaccinated and unvaccinated animals, indicating that vaccination had not ameliorated early changes in clinical parameters following SARS-CoV-2 challenge. Similarly, in terms of virus shedding and the detection of viral replication in nasal and pharyngeal swab samples, evidence of viral RNA and sgRNA was detected in swabs collected from aerosol BCG-vaccinated and unvaccinated animals at comparable levels. Furthermore, equivalent titres of viral RNA and sgRNA were detected in BAL samples collected from vaccinated and unvaccinated animals 6–8 days after SARS-CoV-2 challenge, and in lung, tonsillar, and tracheal samples collected at necropsy, indicating that aerosol BCG vaccination had not enhanced viral clearance during the initial period following challenge, nor altered the prevalence of viral replication. Similarly, the pattern of pulmonary disease measured using in-life CT imaging and histopathological analyses applied to tissue sections postmortem was similar to previous reports in this species (28, 30, 32), with moderate inflammatory changes affecting airways and variable patchy areas of acute pneumonia. There were no prominent differences in the severity of pathological changes noted between BCG-vaccinated and unvaccinated animals indicating that aerosol BCG vaccination did not reduce the occurrence of disease induced by a stringent SARS-CoV-2 challenge within the initial 6–8 days.

Despite the absence of severe COVID-19 disease, many of the immunological features measured in macaques were common with those observed in COVID-19 patients. This included the occurrence of lymphocytopenia (41, 42) which was observed in both BCG-vaccinated and unvaccinated animals transiently following SARS-CoV-2 challenge, and the detection of elevated titres of pro-inflammatory cytokines and chemokine signalling molecules in the serum (43, 44). However, there were also clear indications that aerosol BCG vaccination influenced the immune system and the subsequent immune response to SARS-CoV-2 challenge. This was apparent as a rapid increase in CD14+ classical monocyte populations observed in aerosol BCG-vaccinated animals 1 day after SARS-CoV-2 challenge but not seen in unvaccinated animals. As trained innate immune mechanisms are known to be enacted by epigenetically primed monocyte populations (14, 45), it is likely that this observation is indicative of aerosol BCG vaccination-primed trained immunity. Further evidence of trained immune mechanisms were apparent in the profile of secreted biomarkers measured in serum samples where, although significant increases in biomarker titres were not measured following aerosol BCG vaccination alone, immunisation appeared to alter the production of several proinflammatory and immunomodulatory analytes, as well as cytokines involved in induction of the antiviral immune response following SARS-CoV-2 challenge. Informatively, cytokines such as IL-1β, TNFα, and IL-6, which are known to be central to the innate cellular immune response and are therefore commonly used as surrogate markers of trained innate immunity (10, 11, 46), were detected at an earlier time point post challenge and at concentrations that exceeded titres measured in unvaccinated animals. The role of IL-6, IL-1β, and TNFα in those suffering with COVID-19 is somewhat contradictory, with conflicting reports of these markers being linked to both mild disease and more severe cases typified by the occurrence of an inflammatory syndrome and a dysregulated proinflammatory response (42). However, severe disease has also been linked with delayed immune recognition of infection and the delayed onset of innate and adaptive antiviral immune responses, potentially attributable to coronavirus virulence mechanisms that facilitate the creation of favourable conditions for viral replication and increase the severity of immunopathology (46, 47). Therefore, the rapid induction of inflammatory and immunomodulatory signalling we observed indicates that aerosol BCG vaccination had primed the cellular immune system to circumvent such virulence strategies, which may help to avert the immune dysregulation seen during the later stages of SARS-CoV-2 infection in patients afflicted by severe COVID-19. The early detection of IL-12, IFNα, and IL-2 in vaccinated macaques, cytokines associated with onset of the adaptive antiviral response that have also been linked with asymptomatic and mild disease (49), is a further encouraging sign that aerosol BCG vaccination could provide benefit in individuals prone to develop COVID-19.

Aerosol BCG vaccination induced mycobacteria-specific cell-mediated immune responses, as measured by IFN-γ ELISPOT assays applied to PBMC and tissue-derived MNCs. By contrast, SARS-CoV-2-specific IFN-γ-producing cells were only detected following SARS-CoV-2 challenge, and at equivalent frequencies in vaccinated and unvaccinated animals. Similarly, aerosol BCG vaccination did not induce anti-spike, -RBD, or -NP cross-reactive IgG antibody or increase SARS-CoV-2 neutralising antibody titres. We therefore found little evidence to suggest that aerosol BCG vaccination induced SARS-CoV-2-specific cross-reactive cellular or antibody-mediated immunity, or advanced the onset of the cellular or humoral adaptive immune response following challenge. This indicates that any potential cross-protective effects induced by BCG vaccination are likely to be mediated through trained innate immune mechanisms rather than the recognition of homologous antigens or peptide sequences (13). However, the expansion and activation of circulating Vδ2 T-cell populations, which was associated with reduced disease severity following SARS-CoV-2 challenge, also supports the view that enrolment of unconventional T-cells by BCG vaccination may be beneficial in reducing COVID-19 severity (12).

In conclusion, aerosol BCG vaccination induced mycobacterial antigen-specific cellular immune responses, but there was no indication that vaccination induced, or led to, enhanced induction of SARS-CoV-2 cross-reactive adaptive cellular or humoral immunity. However, the early and enhanced production of proinflammatory and immunoregulatory cytokines detected in serum samples and the detection of increased circulating monocytes and activated γδ T-cell populations in vaccinated animals following SARS-CoV-2 challenge indicates that aerosol BCG vaccination had induced trained innate immune mechanisms and primed unconventional T-cell populations. Nevertheless, readouts of viral shedding, viral load, and challenge induced pathology showed no sign of improvement in vaccinated animals, suggesting that aerosol BCG vaccination does not enhance the initial clearance of virus after high-dose SARS-CoV-2 challenge. However, as manifestations of severe COVID-19 in susceptible patients, which are not replicated by the macaque model, are associated with delayed and dysregulated immune responses during the weeks following infection, it remains possible that the heterologous immune mechanisms induced by BCG vaccination may help to moderate severe COVID-19 disease in susceptible individuals. Further preclinical investigations in animal model species with greater susceptibility to SARS-CoV-2 infection and COVID-19 (32) are required to explore potential cross-protective effects induced by aerosol delivered BCG vaccination, although the findings of the numerous randomized controlled clinical trials currently underway will provide vital evidence as to the value of BCG vaccination as a non-specific prophylactic or therapeutic strategy against the COVID-19 pandemic.

## 4 Materials and Methods

### 4.1 Rhesus Macaques

Twelve rhesus macaques of Indian origin (*Macaca mulatta*) obtained from a UK-based breeding unit were used in the study. Study groups comprised three males and three females, and all were adults aged 2–4 years and weighing between 3.73 and 5.52 kg at the time of challenge. Animals were housed as described previously (28). All experimental work was conducted under the authority of a UK Home Office-approved project licence that had been subject to local ethical review at PHE Porton Down by the Animal Welfare and Ethical Review Body (AWERB).

### 4.2 Aerosol BCG Vaccination

Animals were randomly assigned to unvaccinated and aerosol BCG-vaccinated groups. Sedated animals were exposed to aerosolised BCG Danish strain 1331 (AJ Vaccines, Denmark) using an Omron MicroAir mesh nebuliser (Omron Healthcare United Kingdom Ltd., Milton Keynes, United Kingdom) as described previously (23, 24). Vaccination dose was selected to be approximately equivalent to a standard adult intradermal dose after the expected losses in viable BCG titre associated with the aerosol delivery process were taken into account (24). BCG vaccine was prepared by adding 0.55 ml PBS to each vaccine vial and multiple vials pooled to provide a standardised solution containing approximately 1 × 10^7^ CFU in the 1-ml volume delivered by aerosol.

### 4.3 Virus Stocks and Cells

SARS-CoV-2 Victoria/01/2020 was provided by The Doherty Institute, Melbourne, Australia, at P1 (50) and passaged twice in Vero/hSLAM cells (ECACC 04091501). Briefly, confluent monolayers of hSLAM cells were infected at an approximate multiplicity of infection of 0.0005 for 60 min in minimum essential medium (described below) containing no FBS at 37°C. The flasks were then filled with medium supplemented with 4% heat-inactivated FBS (Sigma-Aldrich, Dorset, UK). Virus was harvested 72 h after infection by removal of any remaining attached cells with sterile 5-mm borosilicate glass beads and clarification of the cell/media supernatant by centrifugation at 1,000*g* for 10 min, followed by dispensing and storage at ≥−65°C. Whole-genome sequencing was performed, on the challenge isolate, using both nanopore and Illumina techniques as described previously (28). Virus titre was determined by plaque assay on Vero/E6 cells (ECACC 85020206). Cell cultures were maintained at 37°C in minimum essential medium (Life Technologies, California, USA) supplemented with 10% FBS (Sigma-Aldrich, Dorset, UK) and 25 mM HEPES (Life Technologies). All Vero/hSLAM cell cultures were also supplemented with geneticin (0.4 μg/ml) (Gibco-Thermo-Fischer Scientific, UK).

### 4.4 SARS-CoV-2 Challenge

Prior to challenge, macaques were sedated by intramuscular injection ketamine hydrochloride (Ketaset, 100 mg/ml, Fort Dodge Animal Health Ltd., UK; 10 mg/kg). SARS-CoV-2 Victoria/01/2020 was prepared as described previously (28). Macaques were challenged with a target dose of 5.0 × 10^6^ plaque-forming units (PFU) delivered by the intratracheal route (2 ml) and intranasal instillation (1 ml total, 0.5 ml to each nare).

### 4.5 Quantification of Viral Shedding and Viral Load

SARS-CoV-2 viral RNA and subgenomic (sg)RNA was quantified in nasal swab, pharyngeal swabs, and bronchoalveolar lavage samples as well as tissue homogenates prepared from lung, tonsillar, and tracheal tissue samples as previously described (29).

### 4.6 Cell Isolation and Interferon-Gamma (IFN-γ) ELISpot Assay

Peripheral blood mononuclear cells (PBMC) and tissue derived mononuclear cells (MNC) were isolated from heparin anticoagulated blood or tissue samples collected during necropsy using standard methods (28). An IFN-γ ELISpot assay was performed as described previously (28) by stimulating 2 × 10^5^ PBMCs, lung mononuclear cells (MNC) or splenocytes using Purified Protein Derivative (PPD, 10 µg) (AJ Vaccines, Denmark), PPD of *Mycobacterium avium* (PPD-A, 10 µg) (NIBSC, UK), rhesus cytomegalovirus (CMV) peptide pool (2.5 µg) (JPT peptides, Germany), SARS-CoV-2 spike protein megapools (MP1, MP2, MP3 that span the whole spike protein) (1.7 µg) (Mimotopes, Australia), and complete SARS-CoV-2: spike (S), membrane protein (M), and nucleocapsid (N) peptide pools (1 μg/ml each, all Miltenyi Biotec, Germany). Samples from each animal were analysed on at least two occasions prior to aerosol BCG vaccination or SARS-CoV-2 challenge and the results averaged to provide a baseline value designated as week -1 graphically (**Figure 2**). Mann–Whitney U-tests were used to compare data obtained from the two groups at each time point, and Wilcoxon paired tests were used to compare each time point to the averaged baseline result within each group.

### 4.7 Immunophenotyping

Whole-blood immunophenotyping assays were performed using 50 µl of heparinised blood incubated for 30 min at room temperature with optimal dilutions of the following antibodies: anti-CD3-AF700, anti-CD4-APC-H7, anti-CD8-PerCP-Cy5.5, anti-CD95-Pe-Cy7, anti-CD14-PE, anti-HLA-DR-BUV395, anti-CD25-FITC (all from BD Biosciences, Oxford, UK); anti-CD127-APC (eBioscience); anti-γδ-TCR-BV421, anti-CD16-BV786, anti-PD-1-BV711, anti-CD20-PE-Dazzle (all from BioLegend); and amine reactive fixable viability stain red (Life Technologies), all prepared in brilliant stain buffer (BD Biosciences). Erythrocyte contamination was removed using a Cal-lyse reagent kit as per the manufacturer’s instructions (Thermo Fisher scientific). BD CompBeads (BD Biosciences) were labelled with the above fluorochromes for use as compensation controls. Following antibody labelling, cells and beads were fixed in a final concentration of 4% paraformaldehyde solution (Sigma-Aldrich, Gillingham, UK) prior to flow cytometric acquisition.

Cells were analysed using a five-laser LSRII Fortessa instrument (BD Biosciences), and data were analysed using FlowJo (version 10, Treestar, Ashland, USA). Immediately prior to flow cytometric acquisition, 50 µl of Trucount bead solution (Beckman Coulter) was added to each sample. Leukocyte populations were identified using a forward scatter-height (FSC-H) versus side scatter-area (SSC-A) dot plot to identify the lymphocyte, monocyte, and granulocyte populations, to which appropriate gating strategies were applied to exclude doublet events and non-viable cells. Lymphocyte subpopulations, including T-cells, NK cells, NKT cells, and B cells, were delineated by the expression pattern of CD3, CD20, CD95, CD4, CD8, CD127, CD25, and CD16 and the activation and inhibitory markers HLA-DR and PD-1 (**Supplementary Figure 10**). GraphPad Prism (version 8.0.1) was used to generate graphical representations of flow cytometry data. An additional whole-blood immunophenotyping panel was utilised to specifically interrogate gd T-cell subsets Vδ1 and Vδ2 and their phenotypes using the following antibodies: anti-CD3-AF700, anti-CCR5-PerCP-Cy5.5 (both from BD Biosciences); anti-Vg9-FITC, anti-Vδ1-PeCy7 (both from Life Technologies); anti-CD69-BV510, anti-NKG2D-APC, anti-CD27-BV711, anti-CD103-BV785, anti-PD-1-BV605, anti-CX3CR1-BV421 (all from BioLegend); anti-CD45RA-APC-Vio770 (Miltenyi Biotec); and anti-TIM3-PE (Bio-Techne). The whole-blood immunophenotyping methodology was carried out as above. Vδ1 and Vδ2 subtypes were delineated *via* the expression of CD3, Vδ1, and Vg9; their activation status *via* CD69 and NKG2D; their memory status *via* CD45RA and CD27; their homing marker expression *via* CCR5, CX3CR1, and CD103; and their regulation/exhaustion expression *via* PD-1 and TIM3 (**Supplementary Figure 11**). For some of the data sets, data from unvaccinated animals were supplemented with that of control group animals from a separate experiment utilising an identical SARS-CoV-2 challenge dose experimental design to mitigate a loss of sample size caused by experimental error in application of the assay (29).

### 4.8 Quantification of Secreted Biomarkers in Serum

Blood samples were collected into serum separation tubes (BD Biosciences) and centrifuged for 10 min at 800 × g; once clots had fully formed, the serum was aspirated and stored at -20°C for later assessment of a range of cytokines, chemokines, and growth factors. Secreted biomarkers in serum samples were quantified using a 30-plex ProcartaPlex bead array assay (Thermo Fischer Scientific, UK) applied according to the manufacturer’s instructions for detection of the following analytes: eotaxin, G-CSF, GM-CSF, IFN-alpha, IFN-gamma, TNF-alpha, IL-10, IL-12p70, IL-13, IL-15, IL-17A, IL-18, IL-1β, IL-1RA, IL-2, IL-23, IL-4, IL-5, IL-6, IL-7, IL-8, IP-10, I-TAC (CXCL11), MCP-1 (CCL2), macrophage inflammatory protein-1α (MIP-1alpha), macrophage inflammatory protein-1α (MIP-1beta) (CCL4), SDF-1alpha (CXCL12), MIG (CXCL9), CD40-ligand, and BLC (CXCL13). Serum samples were analysed using a Luminex MAGPIX instrument (Luminex Corporation, US) equipped with the xPONENT 4.2 software package. Standard curves were generated in duplicate and used to interpolate the concentration of each analyte. All data below the limit of detection specified by the kit manufacturer were assigned a zero value.

### 4.9 IgG ELISA

Recombinant anti-SARS-CoV-2-spike protein, -spike protein receptor binding domain (RBD), and -nucleocapsid protein IgG titres were quantified in serum samples by ELISA as previously described (28).

### 4.10 SARS-CoV-2 Neutralisation Assays

Plaque reduction neutralisation assays were performed using macaque serum samples as described previously for human sera (36). Briefly, neutralising virus titres were measured in heat-inactivated (56°C for 30 min) serum samples. SARS-CoV-2 Victoria/01/2020 was diluted to a concentration of 1.4 × 10^3^ pfu ml^-1^ (70 pfu in 50 ml) and mixed 50:50 in 1% FCS/MEM with doubling serum dilutions from 1:10 to 1:320 in a 96-well V-bottomed plate. The plate was incubated at 37°C in a humidified box for 1 h to allow the antibody in the serum samples to neutralise the virus. The neutralised virus was transferred into the wells of a washed 96-well plate containing virus-susceptible VERO/E6 cells, allowed to adsorb at 37°C for a further hour, and overlaid with 1% CMC in 10% FCS/MEM (Merck, UK). After a 24-h incubation at 37°C in a humified box, the plates were formaldehyde-fixed before infected cell foci were detected by immunostaining with anti-SARS-CoV-2 RBD spike protein (Sino Biological, China), a polyclonal rabbit horseradish peroxidase conjugate (Invitrogen, UK), and TrueBlue Substrate (SeraCare, MA, US). The percentage reduction of foci in serum compared to virus only control was calculated using SoftMax Pro v7.0.3 GxP and neutralisation titres (ND50) reported as the serum dilution that neutralized 50% of the virus foci.

### 4.11 Histopathology

The following samples from each rhesus macaque were sampled during necropsy, fixed in 10% neutral-buffered formalin, processed to paraffin wax and 4-µm-thick sections, cut, and stained with haematoxylin and eosin (HE): left cranial and caudal lung lobes, trachea, larynx, mediastinal lymph node, tonsil, spleen, liver, kidneys (both), duodenum, and colon. For the lung, three sections from each left lung lobe were sampled from different locations: proximal, medial and distal to the primary lobar bronchus. Stained slides were digitally scanned using a Hamamatsu S360 digital slide scanner and examined using ndp.view2 software (c2.8.24). Two qualified veterinary pathologists examined the tissues independently and were blinded to treatment and group details and the slides randomised prior to examination to prevent bias. A semiquantitative scoring system previously developed by our group was used to compare the severity of the lung lesions for each individual animal and among groups (28). The scores for each histopathological parameter were calculated as the average of the scores observed in the six lung tissue sections evaluated per animal.

In addition, tissue sections were stained using the *in situ* hybridisation (ISH) RNAscope technique to label the SARS-CoV-2 virus RNA S-gene. Briefly, tissues were pretreated with hydrogen peroxide for 10 min (room temperature), target retrieval for 15 min (98°C–101°C), and protease plus for 30 min (40°C) (Advanced Cell Diagnostics). A V-nCoV2019-S probe (Cat No. 848561, Advanced Cell Diagnostics) was incubated on the tissues for 2 h at 40°C. Amplification of the signal was carried out following the RNAscope protocol using the RNAscope 2.5 HD Detection Kit – Red (Advanced Cell Diagnostics).

Nikon NIS-Ar software was used to perform digital image analysis to quantify the presence of viral RNA in lung sections calculating the total area of the lung section positive for viral RNA.

### 4.12 In-Life Imaging of Macaques by Computed Tomography

CT scans were collected 4 weeks before vaccination and 5 days after challenge. CT imaging was performed on sedated animals using a 16-slice LightSpeed CT scanner (General Electric Healthcare, Milwaukee, WI, USA) in both the prone and supine positions to assist the differentiation of pulmonary changes at the lung bases caused by gravity dependant atelectasis, from ground-glass opacity caused by SARS-CoV-2. All axial scans were performed at 120 kVp, with Auto mA (ranging between 10 and 120), and were acquired using a small scan field of view. Rotation speed was 0.8 s. Images were displayed as an 11-cm field of view. To facilitate full examination of the cardiac and pulmonary vasculature, lymph nodes, and extrapulmonary tissues, Niopam 300 (Bracco, Milan, Italy), a non-ionic, iodinated contrast medium was administered intravenously (IV) at 2 ml/kg body weight and scans were collected immediately after injection and 90 s from the mid-point of injection.

Scans were examined by a medical consultant radiologist with expertise in respiratory diseases, including in non-human primates (28, 51). Disease features characteristic of COVID-19 in humans were evaluated including ground-glass opacity (GGO), consolidation, crazy paving, nodules, and peri-lobular consolidation; distribution: upper, middle, lower, central 2/3, bronchocentric); pulmonary embolus; and the extent of any abnormalities estimated (<25%, 25%–50%, 51%–75%, 76%–100%), whilst being blinded to the animals’ clinical status to avoid the potential for bias.

### 4.13 CT Score System

To provide the power to discriminate differences between individual NHPs with low disease volume (i.e., <25% lung involvement), a scoring system was applied in which scores were attributed for possession of abnormal features characteristic of COVID in human patients (COVID pattern score) and for the distribution of features through the lung (Zone score). The COVID pattern score was calculated as sum of scores assigned for the number of nodules identified, and the possession and extent of GGO and consolidation according to the following system: Nodule(s): Score 1 for 1, 2 for 2 or 3, 3 for 4 or more; GGO: each affected area was attributed with a score according to the following: Score 1 if area measured < 1 cm, 2 if 1 to 2 cm, 3 if 2–3 cm, 4 if > 3 cm; and scores for each area of GGO were summed to provide a total GGO score; Consolidation: each affected area was attributed with a score according to the following: 1 if area measured < 1 cm, 2 if 1 to 2 cm, 3 if 2–3 cm, 4 if > 3 cm. Scores for each area of consolidation are summed to provide a total consolidation score. To account for estimated additional disease impact on the host of consolidation compared to GGO, the score system was weighted by doubling the score assigned for consolidation. To determine the zone score, the lung was divided into 12 zones and each side of the lung divided (from top to bottom) into three zones: the upper zone (above the carina), the middle zone (from the carina to the inferior pulmonary vein), and the lower zone (below the inferior pulmonary vein). Each zone was further divided into two areas: the anterior area (the area before the vertical line of the midpoint of the diaphragm in the sagittal position) and the posterior area (the area after the vertical line of the mid-point of the diaphragm in the sagittal position). This results in 12 zones in total where a score of one is attributed to each zone containing structural changes. The COVID pattern score and the zone are summed to provide the total CT score.

### 4.14 Statistical Analysis

Statistical comparison of post challenge weight change and viral shedding were based on area under the curve (AUC) values calculated from the day of challenge to a unified 7-day post challenge end point, although measurements may have been collected on days 6, 7, or 8 post challenge. Similarly, for IFN-γ ELISpot, ELISA IgG titres, neutralising antibody titres, whole-blood immunophenotyping and secreted biomarker profiles, and parameters quantified in samples collected at necropsy were plotted as one point for graphical purposes although samples may have been collected at either 6, 7, or 8 days post challenge. Where appropriate, these unified last data points were used to calculate AUC values for comparative analysis. All data comparisons applied used non-parametric Mann–Whitney U-tests or Wilcoxon matched-pair test as appropriate and were conducted using GraphPad Prism v9.0.0.

## Data Availability Statement

The raw data supporting the conclusions of this article will be made available by the authors, without undue reservation.

## Ethics Statement

The animal study was reviewed and approved by the UK Health Security Agency Ethics Review Committee.

## Author Contributions

ADW, MD, MBS, MC HMS, WC, JH, and SS contributed to the conception and design of the work. ADW, LS, CS, ALM, KoG, AM, CC, MM, SL, HH, KaG, RW, AH, KB, SF, LH, CK, ER, YH, FG, FJS, and MD contributed to the methodology, data acquisition, analysis and interpretation, or administration of the work. AW, LS, CS, AM, JS, ER, and SS wrote the manuscript, and all authors provided critical review. All authors contributed to the article and approved the submitted version.

## Funding

The studies described herein were funded by grants from the Coalition for Epidemic Preparedness Innovations (CEPI), Public Health England, and the Institute for Cancer Vaccines and Immunotherapy (Registered Charity Number 1080343).

## Conflict of Interest

The authors declare that the research was conducted in the absence of any commercial or financial relationships that could be construed as a potential conflict of interest.

## Publisher’s Note

All claims expressed in this article are solely those of the authors and do not necessarily represent those of their affiliated organizations, or those of the publisher, the editors and the reviewers. Any product that may be evaluated in this article, or claim that may be made by its manufacturer, is not guaranteed or endorsed by the publisher.
