# Supplementary material for: Influence of Aerosol Delivered BCG Vaccination on Immunological and Disease Parameters Following SARS-CoV-2 Challenge in Rhesus Macaques
Source: Front Immunol. 2022 Feb 9;12:801799. doi: 10.3389/fimmu.2021.801799 (PMC8863871; doi:10.3389/fimmu.2021.801799)
Supplement: Supplementary file 1 [file DataSheet_1.pdf]

Supplementary data

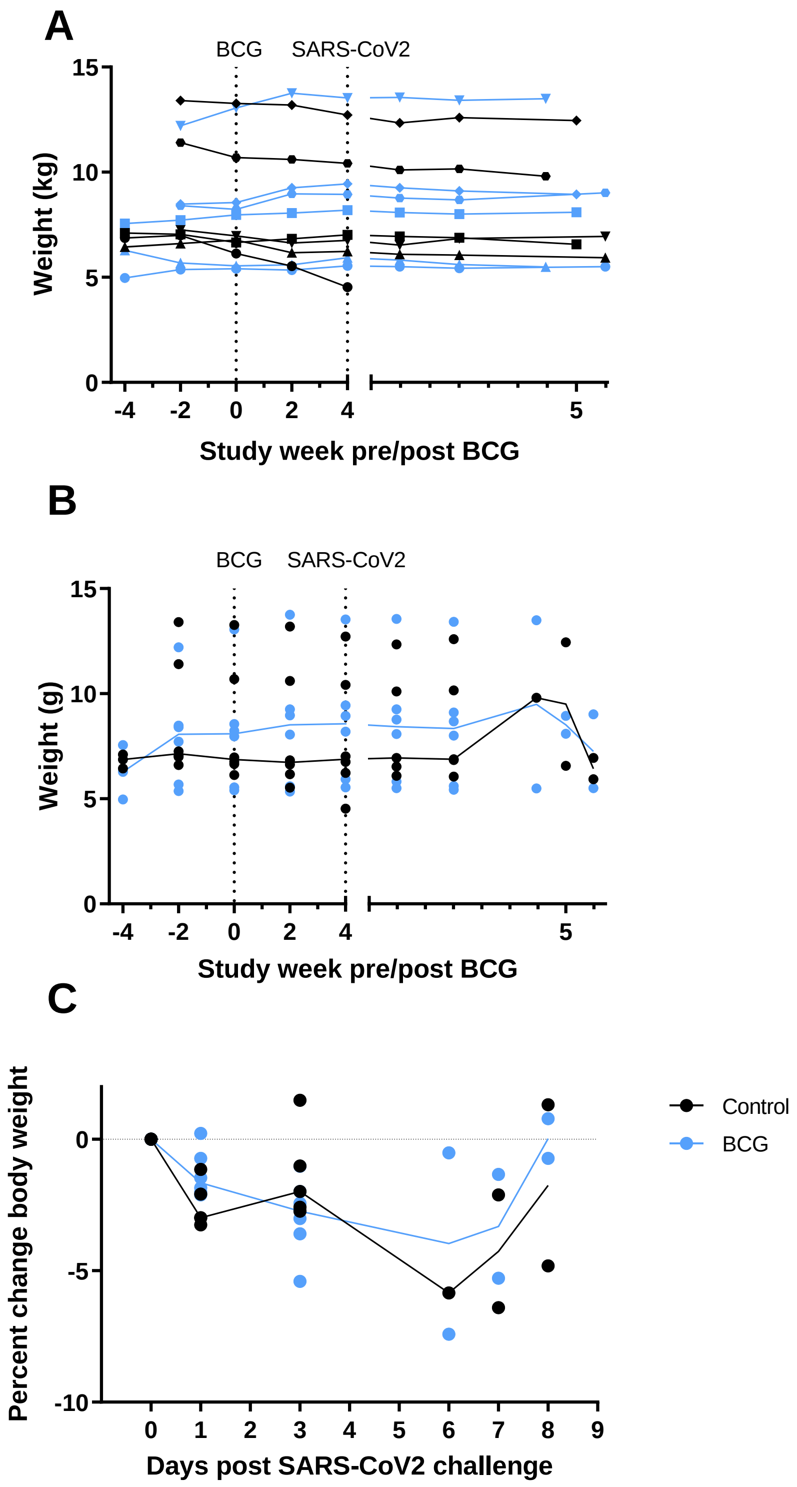

Supplementary figure 1: Body weight measurements recorded pre/post aerosol BCG vaccination and SARS-CoV-2 challenge. Weight measurements recorded from individual animals are shown in Figure A (unvaccinated = black, vaccinated = blue), with group median values indicated in figures B and C.

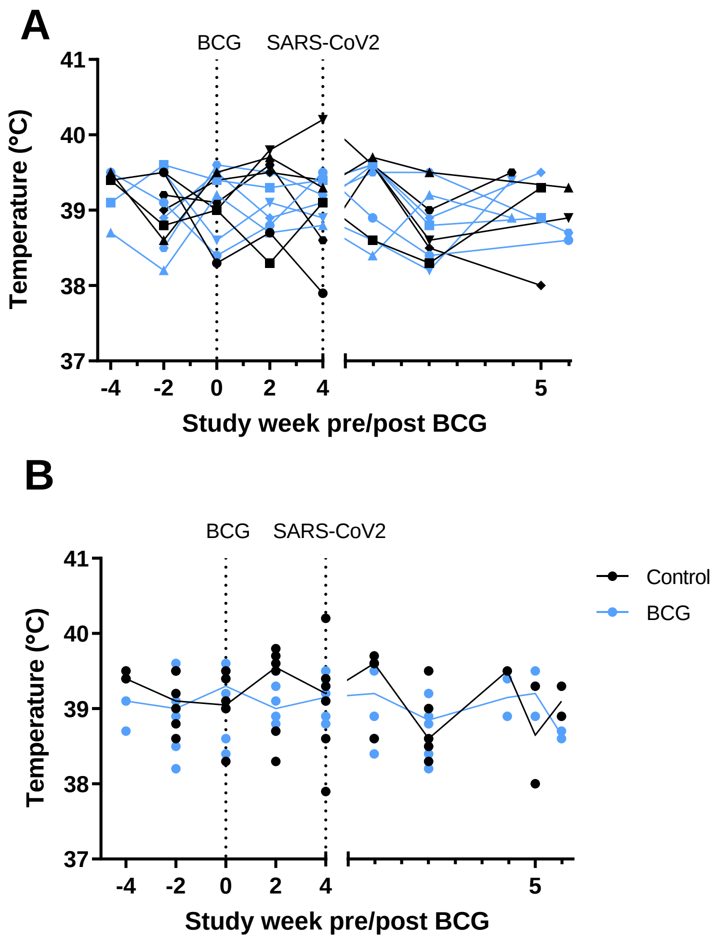

Supplementary figure 2. Body temperature measurements recorded before and after aerosol BCG vaccination and SARS-CoV-2 challenge. Temperature measurements recorded from individual animals are shown in Figure A (unvaccinated = black, vaccinated = blue), with group median values indicated in figure B.

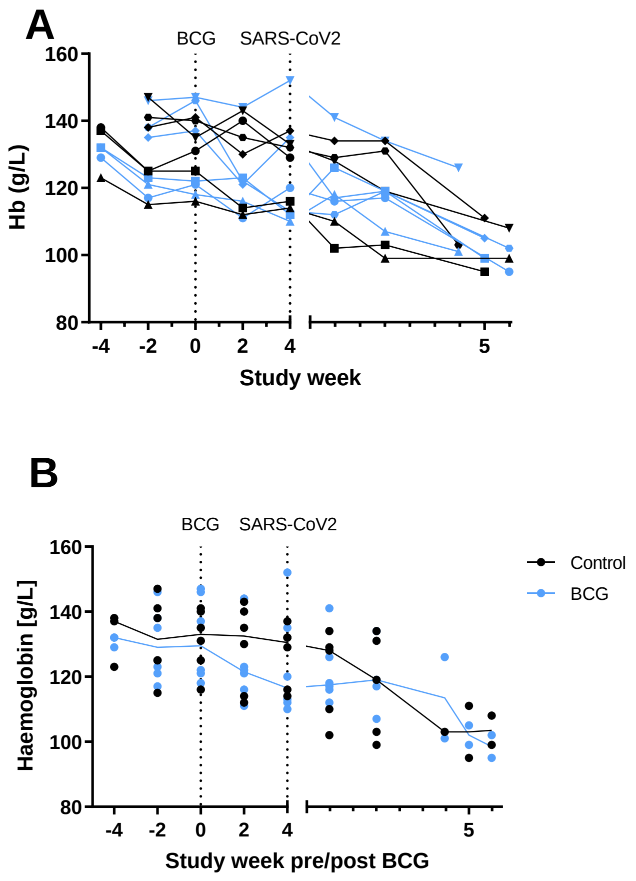

Supplementary figure 3: Red blood cell haemoglobin concentration measurements recorded before and after aerosol BCG vaccination and SARS-CoV-2 challenge. Red blood cell haemoglobin concentration recorded from individual animals are shown in Figure A (unvaccinated = black, vaccinated = blue), with group median values indicated in figure B.

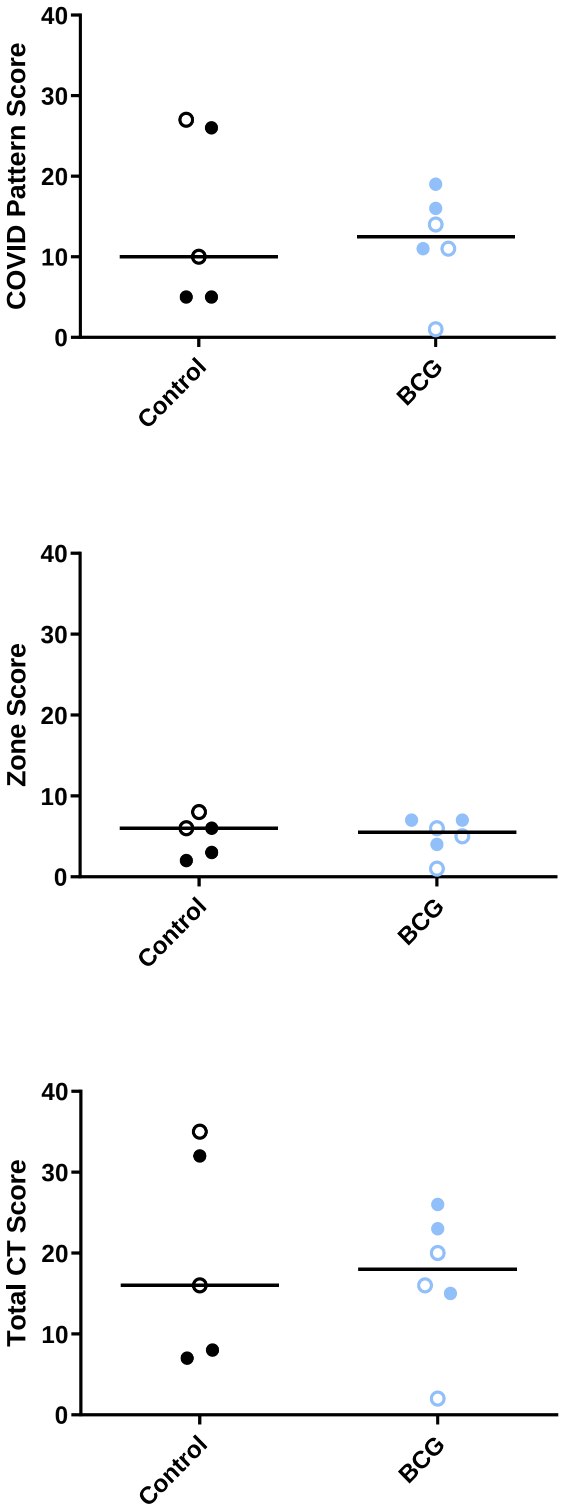

Supplementary figure 4: Graphical representation of the CT scan pulmonary disease burden quantitative score. Bars show the group median. Closed symbols show males, open symbols show females.

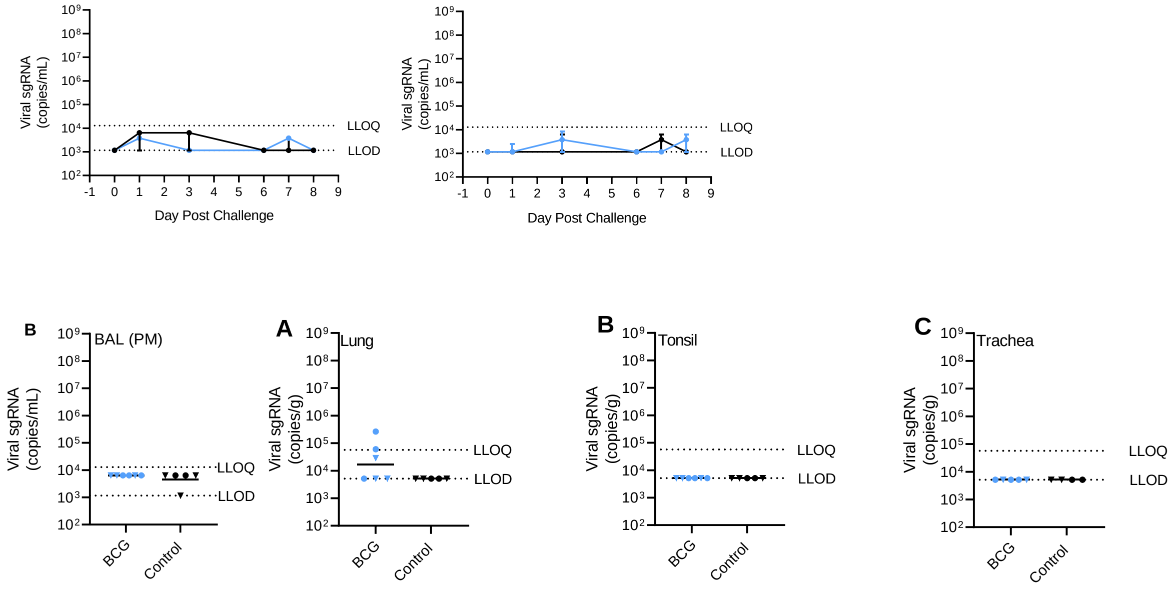

Supplementary figure 5: Viral sgRNA detected in nasal or throat swab samples and in tissues collected at necropsy. Group median titres of viral sgRNA (+/- IQR) detected in A) nasal and B) throat swab samples following SARS-CoV-2 challenge. Lower limits of detection (LLOD) and quantification (LLOQ) are indicated. C - F) Viral sgRNA detected in the BAL(C), Lung (D), tonsil (E)and trachea (F) samples collected at necropsy. Symbols represent viral sgRNA titres recorded from individual animals (females = triangles, males = circles) with group medians indicated.

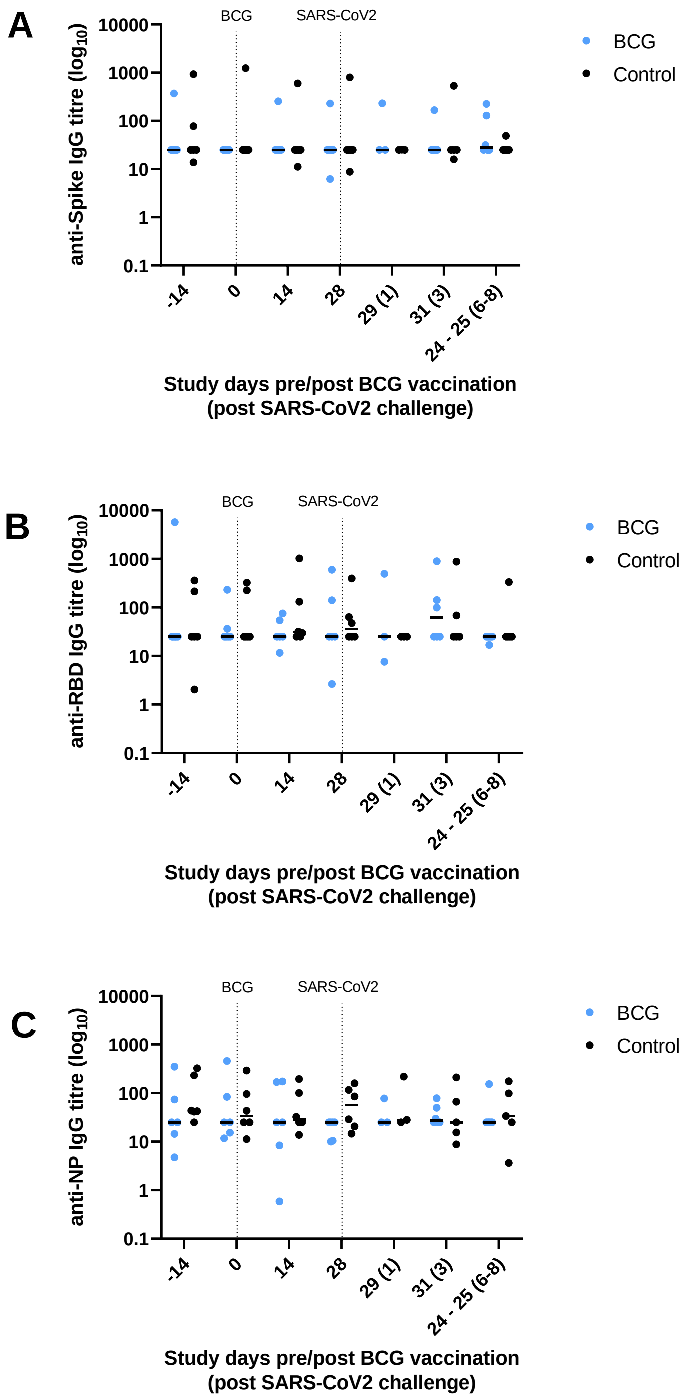

Supplementary figure 6: A) Spike-, B) RBD- and C) NP-specific IgG titres measured in serum before and after aerosol BCG vaccination and SARS-CoV-2 challenge. IgG titres measured in individual animals are shown with group median values indicated.

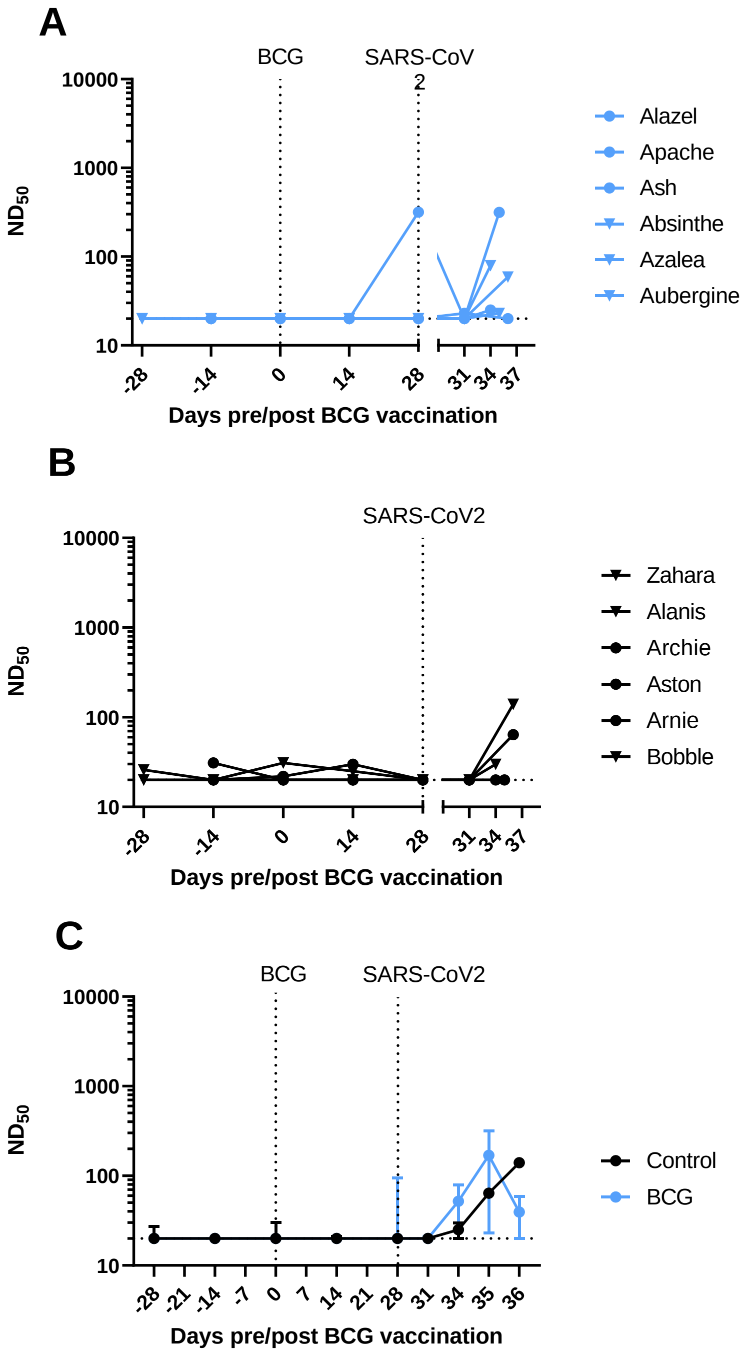

Supplementary figure 7: Neutralising antibody titres measured in serum before and after aerosol BCG vaccination and SARS-CoV-2 challenge. Neutralising antibody titres measured in A) aerosol BCG vaccinated and B) unvaccinated individual animals. Panel C shows group median titres +/- interquartile range. Aerosol BCG vaccination and SARS-CoV-2 challenge are indicated by reference lines.

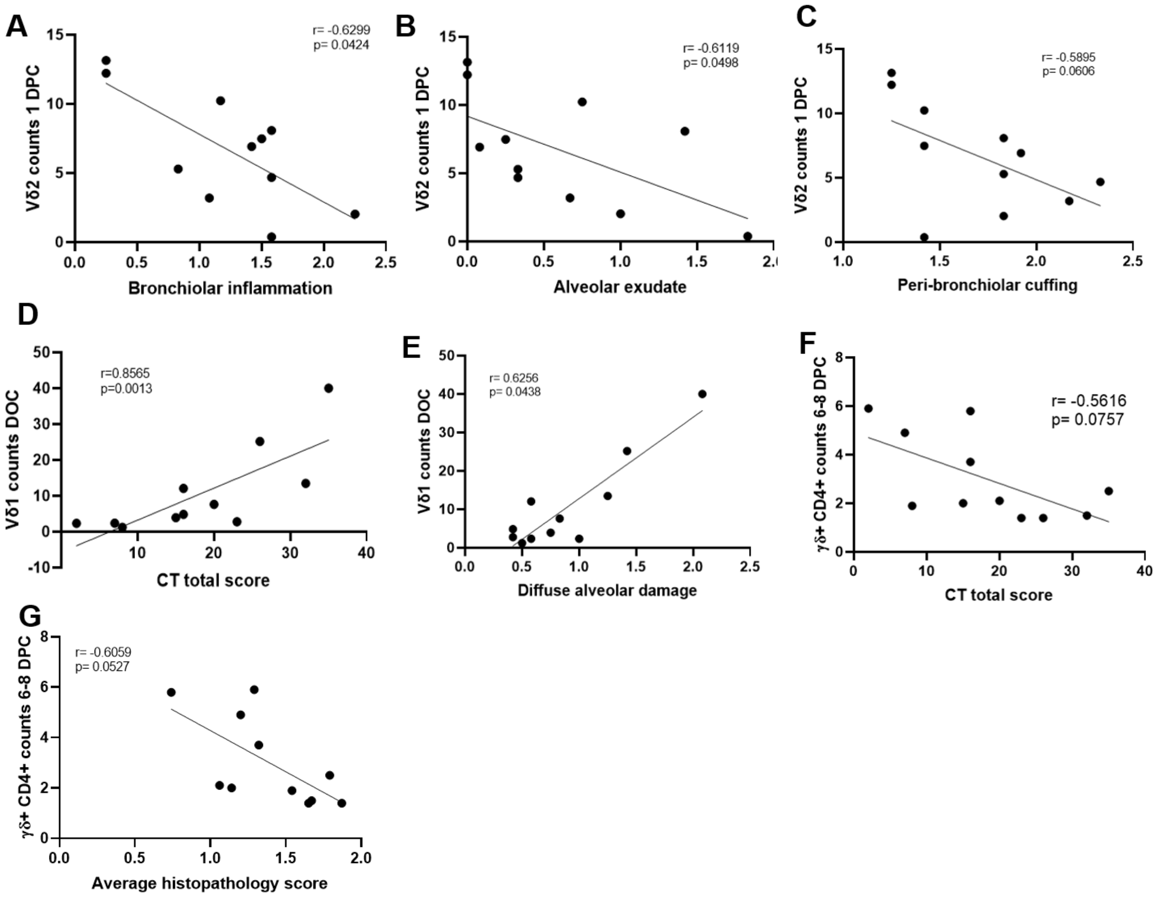

Supplementary figure 8. γδ T cell WBIP correlations with post SARS-CoV-2 challenge disease outcome measures. Correlations between disease outcome measures and whole blood counts of Vδ2+ (A-C), Vδ1+ (D-E) and γδ+ CD4+ (F-G) T cells at: the day of SARS-CoV-2 challenge (DOC); one day post challenge (1 DPC); and 6-8 days post challenge (6-8 DPC), were interrogated via Spearman rank correlation tests. Both vaccinated and unvaccinated animals are included in this analysis.

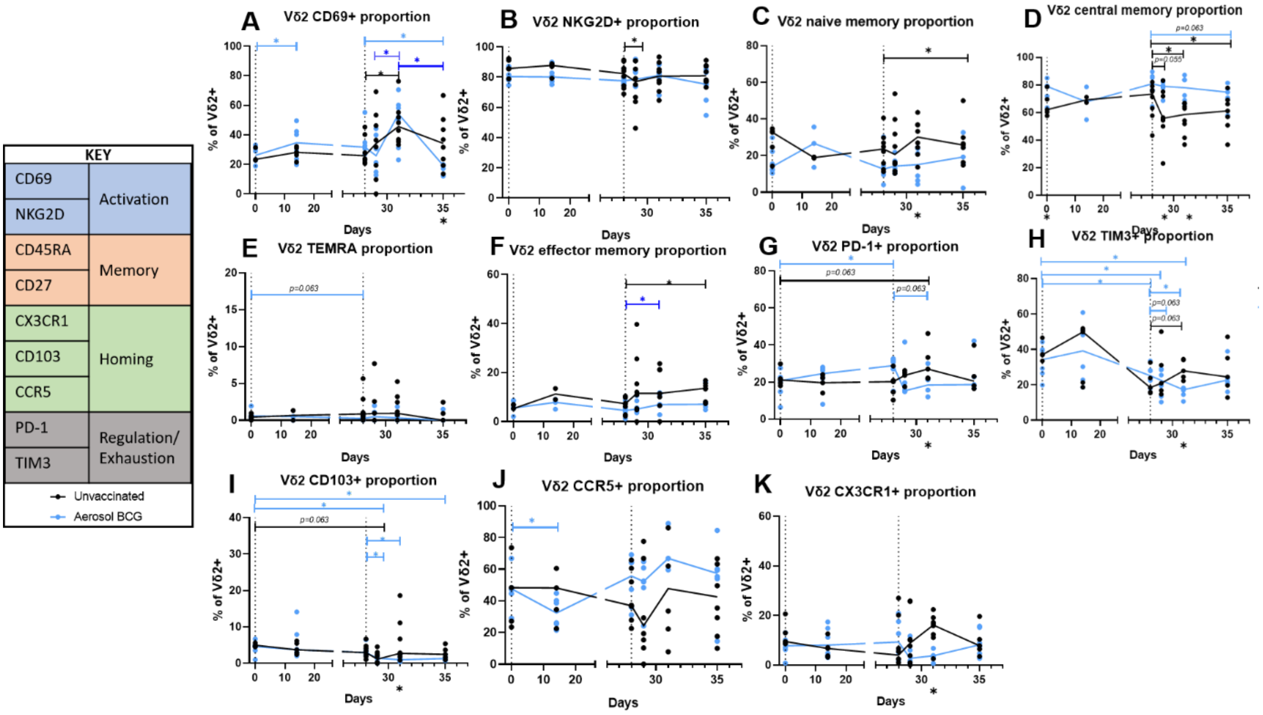

Supplementary figure 9. Vδ2 phenotypes as measured using WBIP before and after aerosol BCG vaccination and SARS-CoV-2 challenge. A-B) Activation, C-F) memory, G-H) regulated/exhausted and I-J) migration/homing phenotypes of Vδ2 T cells were investigated throughout the study. All populations shown as a proportion of Vδ2+ T cells using phenotypic markers indicated in the key. each animal represented by a dot. Light blue = BCG vaccinated, black = unvaccinated. Significant differences determined by Wilcoxon signed-rank test for comparisons within groups (colour coded by group and dark blue = both groups) denoted by asterisks above, and Mann-Whitney U-test for comparisons between groups are denoted by asterisks below X-axis. **p* = 0.05, ***p* = 0.01, ****p* = 0.001.

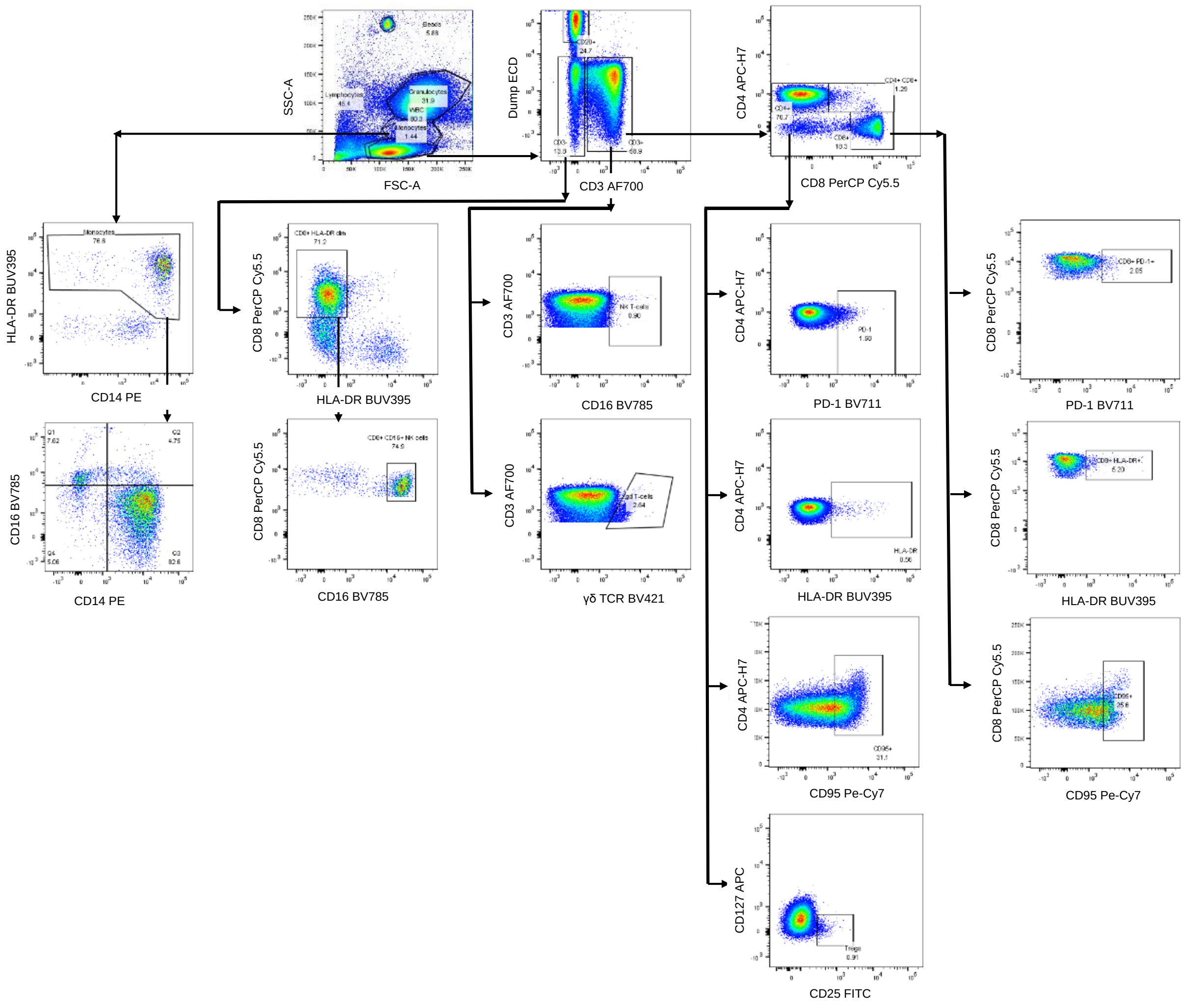

Supplementary figure 10: Whole blood immunophenotyping flow cytometry gating strategy. Leukocyte populations were identified using a forward scatter-height (FSC-H) versus side scatter-area (SSC-A) dot plot to identify the lymphocyte, monocyte and granulocyte populations, to which appropriate gating strategies were applied to exclude doublet events and non-viable cells. Lymphocyte sub populations, including T-cells, NK-cells, NKT-cells and B-cells were delineated by the expression pattern of CD3, CD20, CD95, CD4, CD8, CD127, CD25, CD16 and the activation and inhibitory markers HLA-DR and PD-1.

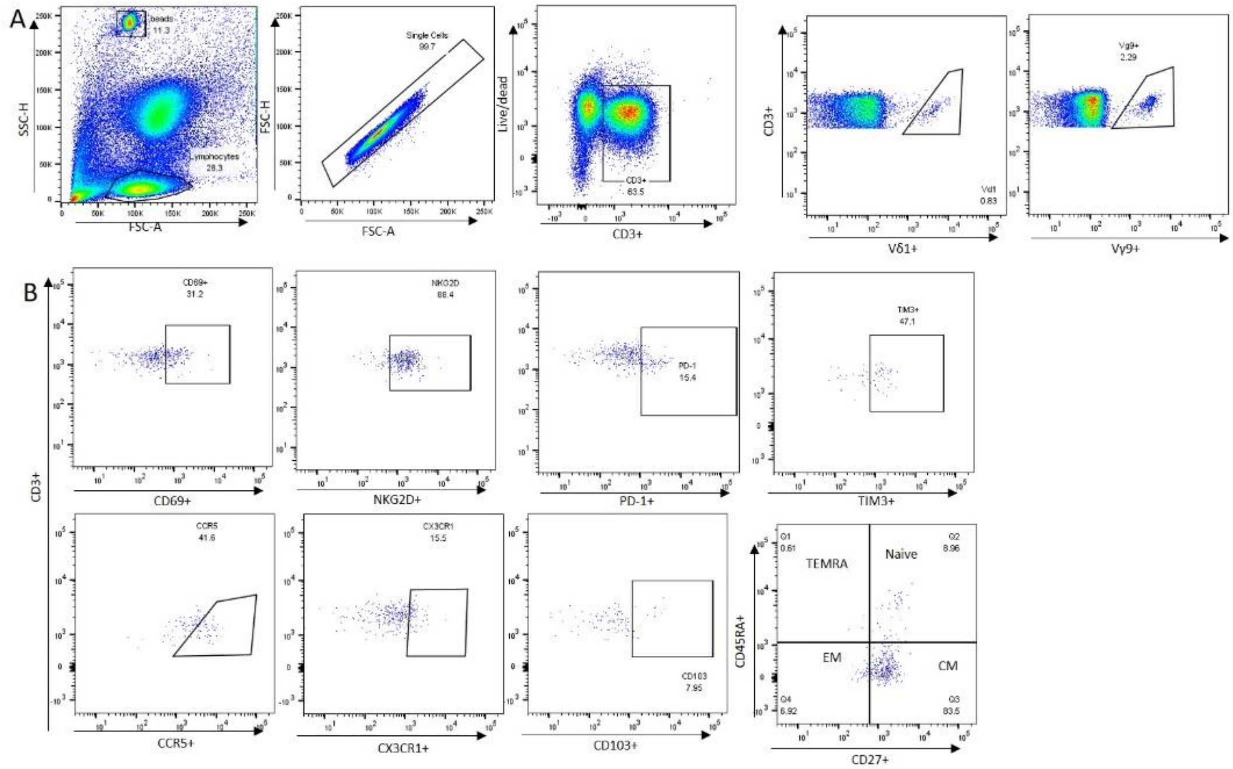

Supplementary figure 11: gating strategy for whole blood immunophenotyping of γδ T cells. A) Lymphocytes were gated on from the whole blood using side scatter and forward scatter, and further gated on singe cells and live, CD3+ cells. From the CD3+ population Vδ1 and Vδ2 cells were gated on using Vδ1 and Vγ9 antibodies respectively. Cell counts were determined through the use of Flow-Count Fluoropheres. B) Vδ2 cells were gated on and phenotypic markers were used to look at activation (CD69, NKG2D), regulation/exhaustion (PD-1, TIM3), homing/tissue residency (CCR5, CX3CR1, CD103) and memory populations (CD45RA, CD27).
